# Mental health trajectories in university students across the COVID-19 pandemic: findings from the Student Wellbeing at Northern England Universities prospective cohort study

**DOI:** 10.3389/fpubh.2023.1188690

**Published:** 2023-07-17

**Authors:** Lewis W. Paton, Paul A. Tiffin, Michael Barkham, Bridgette M. Bewick, Emma Broglia, Lisa Edwards, Louise Knowles, Dean McMillan, Paul N. Heron

**Affiliations:** ^1^Hull York Medical School, University of York, York, United Kingdom; ^2^Department of Health Sciences, University of York, York, United Kingdom; ^3^Department of Psychology, University of Sheffield, Sheffield, United Kingdom; ^4^School of Medicine, University of Leeds, Leeds, United Kingdom; ^5^Student Mental Health, Counselling and Therapies Service, The University of Sheffield, Sheffield, United Kingdom; ^6^Faculty of Health Studies, University of Bradford, Bradford, United Kingdom

**Keywords:** students, wellbeing, COVID-19, latent trajectories, longitudinal

## Abstract

**Introduction:**

Psychological wellbeing in university students is receiving increased focus. However, to date, few longitudinal studies in this population have been conducted. As such, in 2019, we established the Student Wellbeing At Northern England Universities (SWANS) cohort at the University of York, United Kingdom aiming to measure student mental health and wellbeing every six months. Furthermore, the study period included the COVID-19 pandemic, giving an opportunity to track student wellbeing over time, including over the pandemic.

**Methods:**

Eligible participants were invited to participate via email. Data were collected, using Qualtrics, from September 2019 to April 2021, across five waves (W1 to W5). In total, *n* = 4,622 students participated in at least one wave of the survey. Data collection included sociodemographic, educational, personality measures, and mental health and wellbeing. Latent profile analyses were performed, exploring trajectories of student wellbeing over the study period for those who had completed at least three of the five waves of the survey (*n* = 765), as measured by the Warwick-Edinburgh Mental Wellbeing Scale (WEMWBS).

**Results:**

Five latent profile trajectories of student wellbeing were identified. Of these, the two latent classes with initially higher wellbeing scores had broadly stable wellbeing across time (total *n* = 505, 66%). Two classes had lower initial scores, which lowered further across time (total *n* = 227, 30%). Additionally, a fifth class of students was identified who improved substantially over the study period, from a mean WEMWBS of 30.4 at W1, to 49.4 at W5 (*n* = 33, 4%). Risk factors for having less favourable wellbeing trajectories generally included identifying as LGBT+, self-declaring a disability, or previously being diagnosed with a mental health condition.

**Conclusion:**

Our findings suggest a mixed picture of the effect of the COVID-19 pandemic on student wellbeing, with a majority showing broadly consistent levels of wellbeing across time, a smaller but still substantial group showing a worsening of wellbeing, and a small group that showed a very marked improvement in wellbeing. Those from groups traditionally underrepresented in higher education were most at risk of poorer wellbeing. This raises questions as to whether future support for wellbeing should target specific student subpopulations.

## 1. Introduction

There are concerns about the mental health of university students (1), including suicidal ideation and behaviour (2). The prevalence of mental health conditions amongst students appears to be increasing. For example, a fivefold increase in first year students disclosing a mental health problem to their university has previously been reported (3). There has also been a dramatic increase in the number of students seeking counselling over the past decade (3, 4). Moreover, the prevalence of mental health problems amongst young adults, who represent the majority of the student population, has also increased from 15 to 19% between 1993 and 2014 (5). The rate of student deaths by suicide, however, has been decreasing, from 4.6 per 100,000 students in 2016/2017, to 3.0 per 100,000 in 2019/2020 (6). This, however, is likely at least partly explained by delays in the Coroners courts due to the COVID-19 pandemic (7).

Widening participation in higher education has led to a UK student population that is more representative of the general population. For example, more people from disadvantaged socioeconomic backgrounds are now attending university (8). It has previously been suggested that the changing student population will increase the portion of the student population at risk of mental health problems (9). It is not, however, clear if the recent increases in student support service use reflects this specific scenario, a deterioration in the psychological wellbeing of student populations, a tendency towards increasing help-seeking behaviour, larger student populations, or a combination of all these factors.

However, there is currently a dearth of evidence that could facilitate an understanding of long-term university student mental health trends. The COVID-19 pandemic impacted on those studying at higher education institutions in multiple ways, such as online teaching and assessments (10) and additional economic stressors, such as students paying rent on unused accommodation and fewer opportunities for part-time paid work. The findings from three surveys conducted in November 2020 reported that more than half of students reported a deterioration in their mental health and wellbeing as a result of COVID-19 (11). A survey conducted after pandemic restrictions were lifted showed that reported anxiety levels were, on average, higher than those for the pre-pandemic period. Loneliness was also found to be a common problem, although other wellbeing indicators such as happiness and life satisfaction had returned to pre-pandemic levels (12). These findings are broadly in line with the published research into the effects of the pandemic on the UK general population, which have reported a general worsening of mental health and wellbeing symptoms, particularly earlier in the pandemic and in younger people (13–16).

Those from low socioeconomic backgrounds, ethnic minority backgrounds, LGBT+ students, older students, those with poorer social skills, and those with previous or existing mental illnesses, are all vulnerable to risk factors for worse mental health outcomes at university (17, 18). There have also been differences observed across courses: for example, it has previously been observed that medical students are reported to have higher rates of mild depression than those on non-medical courses, although the opposite observation was made for moderate and severe depression (19), whilst social science students have also been observed to be at increased risk of depression (20).

However, the impact of the pandemic is likely to be complex and may have differential effects on different groups. The COVID-19 pandemic had a disproportionate impact on the mental health and wellbeing of a number of groups. For example, women, those with existing mental health conditions, and those from lower socioeconomic backgrounds reported worse outcomes (13, 14, 16). Within university students, a Danish study found that students with lower levels of extraversion experienced a slight increase in mood during COVID-19, whereas greater extraversion was associated with decreased mood (21). Moreover, an Italian study reported cognitive thinking style to be related to mental health outcomes during the pandemic (22). There are also concerns that international students were at greater risk of mental health problems during COVID-19 due to travel restrictions and isolation (23). However, most published studies report qualitative observations (24). Student COVID Insight Surveys have provided quantitative evidence for COVID-19 impacts on mental health though, crucially, they lacked pre-COVID-19 baseline measures that provide relevant context to understand changes over time (11).

A number of longitudinal studies have been published which include contemporaneous pre-COVID-19 baseline measures, allowing for the evaluation of the relative impact of the pandemic. A study of 254 UK undergraduate students reported increased clinically-significant depressive symptoms on the Hospital Anxiety and Depression Scale at 6 months compared to a pre-pandemic baseline, and also significantly decreased wellbeing (25), although the study did not measure the longer-term impacts of the pandemic. A number of European studies have observed deteriorating mental health outcomes, compared to pre-pandemic baselines (26, 27), although these findings are not universal (28). However, findings from these non-UK based studies have low comparability to UK students due to contextual differences in lockdown restrictions.

Accordingly, the longitudinal studies published thus far offer a limited understanding of university student mental health or wellbeing trends in the UK in the ‘recovery’ phase of the pandemic. Moreover, there is likely to be considerable heterogeneity in individuals’ psychological responses to the pandemic in the student body.

The ‘Student Wellbeing At Northern England Universities’ (SWANS) study comprises a series of separate prospective longitudinal cohort studies at a number of universities across the north of England and aims to explore psychological distress and wellbeing in higher education students. The first of these studies was established at the University of York in September 2019, and collects data every six months. To date, no papers have been published from these cohort studies. Additionally, the timeline of data collection provides an opportunity to explore long-term trends in student wellbeing before and during the COVID-19 pandemic. In order to guide future policy and practice developments, it is important both to identify potentially differing mental health trajectories within the student population, and also map this out across the entire pandemic period and beyond, thereby requiring sampling before and after the onset of the pandemic.

The study thus had the following aims:

- To describe the SWANS cohort at the University of York, across the first five waves of data collection and
- To identify, using latent profile analyses (29), latent profile trajectories of student wellbeing across the five waves studied. This should permit the identification of unobserved groups of individuals that are both relatively vulnerable, or relatively resilient, to the impact of a pandemic and the associated societal changes.

## 2. Methods

### 2.1. Design

Data were drawn from the ongoing SWANS study at the University of York, a prospective longitudinal cohort study. This study collects sociodemographic data and validated measures of wellbeing and psychological distress. Starting in September 2019, the SWANS study recruits new participants and collects data every six months.

### 2.2. Ethics approval and consent to participate

The University’s Department of Health Sciences Research Governance Committee provided ethical approval for the study (HSRGC/2019/346/J). Potential participants were referred to a participant information sheet, and were informed that completing the survey was considered to be providing informed consent to the study. Upon completing the survey, participants were invited to provide consent to: (i) be contacted every six months to complete follow-up surveys; (ii) receive invitations to related research; or (iii) be involved in future data linkage studies. At future waves, participants who consented to be contacted for follow-up surveys were sent two emails inviting them to the study in addition to the general invitation emails.

### 2.3. Participants

The target population (approx. *n* = 20,000) were either prospective individuals who had received an offer of a place, or were currently studying, at the University of York, and aged over 18 years. No restrictions were placed on course studied, and both undergraduate and postgraduate students were eligible to participate. Exclusion criteria were those on a leave of absence, on an extension to their studies, attending a single freestanding course, or pre-sessional students.

### 2.4. Recruitment and data collection

Eligible participants were sent two emails inviting them to complete the first wave of the survey at the start of the academic year (W1). Invitation emails were distributed by the University’s student communications department, on behalf of the independent research team. Poster advertisements that referred students to SWANS were also placed on campus and the University’s student webpages.

**Figure 1:**
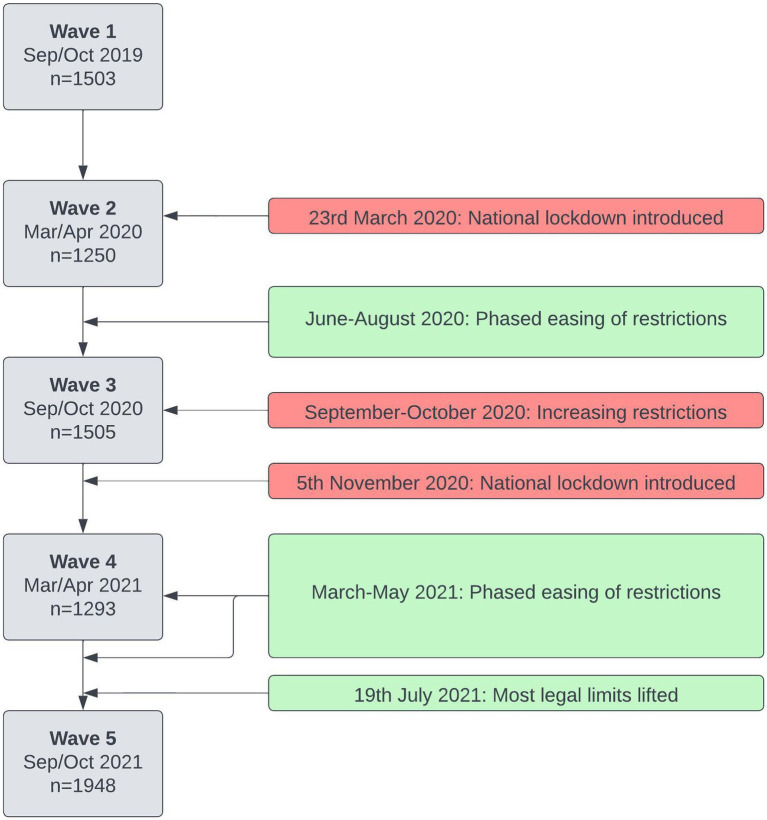
Timeline of data collection in the study, and some key milestones relating to the COVID-19 pandemic in the United Kingdom (31).

Recruitment and data collection occurred over a four-week period. Data were collected using Qualtrics (30). The first wave (W1) occurred in September and October 2019. The described methods were then repeated at six months intervals: wave 2 (W2) was in March and April 2020, wave 3 (W3) in September and October 2020, wave 4 (W4) in March and April 2021, and wave 5 (W5) in September and October 2021. The timeline of data collection in the study, as well as how this aligned with some key milestones relating to the COVID-19 pandemic in the UK (31), is shown in Figure 1.

### 2.5. Measures

#### 2.5.1. Demographic and educational data

The following self-report demographic measures were collected for each participant during each wave:

- *Gender identity*: dichotomised as male or female. Those who reported other gender identities were recorded as missing for analytic purposes.
- *Sexual orientation*: responses available were heterosexual, bisexual, gay man, gay woman/lesbian, or other. For analytic purposes, these categories were dichotomised as heterosexual or LGBT+ [Lesbian, Gay, Bisexual, Transgender, or other sexual identities (+)].
- *Ethnicity:* responses available were White, Black/African/Caribbean/Black British, Asian/Asian British, Mixed/multiple ethnic groups, or other. For analytic purposes, these were dichotomised as White or non-White.
- *Disability:* responses available were no known, specific learning disability, long standing illness or health condition, mental health condition, two or more disabilities, or other. For analytic purposes, these were dichotomised as one or more known disability, or no disability.
- *Parents’ level of education*: responses available were either higher education or not.
- *Index of Multiple Deprivation (IMD)*: for home students, we had IMD data, a national classification of relative deprivation based on home location (32). The index is classified into deciles, with the first decile being amongst the 10% most deprived, and the tenth decile the 10% least deprived in England.

Participants were also asked about their current education status at each wave. We collected faculty of study (Arts and Humanities, Sciences or Social Sciences), level of study (undergraduate or postgraduate), current year of course and whether they were an overseas or home student.

#### 2.5.2. Mental health and wellbeing

At each wave, participants were asked to complete the Warwick-Edinburgh Mental Wellbeing Scale (WEMWBS) (33), as a measure of wellbeing. The WEMWBS comprises 14 items [see electronic supplementary material in Tennant et al. (33)]. Each item is scored from one to five, with higher scores indicating better mental wellbeing. Total WEMWBS scores thus range from 14 to 70. The WEMWBS has been shown to be acceptably reliable and a valid measure of wellbeing in students (34, 35) and has demonstrated high internal consistency (Cronbach’s alpha of 0.89) and test–retest reliability (0.83) in a UK university sample (33).

Participants also completed the General Population Clinical Outcomes in Routine Evaluation (GP-CORE) (36), as a measure of symptoms of psychological distress, designed for use in a general or non-clinical population. The GP-CORE comprises 14 items, with a total score ranging from zero to 56, and higher scores indicating worse outcomes. It has been validated in students (36).

Participants were also asked whether they had ever previously sought help for a mental health problem from a healthcare professional, and similarly whether they had ever received a diagnosis for a mental health problem. In addition, during W4 (March/April 2021), a number of additional validated measures relating to mental health and wellbeing were administered. Current depressive symptoms were measured using the Patient Health Questionnaire-8 (PHQ-8) (37), an eight item scale with total scores ranging from zero to 24. Current anxiety symptoms were measured using the Generalised Anxiety Disorder-7 assessment (GAD-7) (38), a seven item scale with total scores ranging from zero to 21. Loneliness was measured by the UCLA Loneliness Scale (ULS-8) (39), an eight item scale with total scores ranging from eight to 32. In all three scales a higher score indicates worse symptoms. We present these data for completeness.

#### 2.5.3. Personality measure

From W3 (September/October 2020) onwards, participants were asked to complete the Big Five Inventory-2 short form (BFI-2-S) (40). This is a self-report instrument that asks about personality traits (openness, conscientiousness, extraversion, agreeableness and neuroticism). Where students had responded to multiple waves, only the first set of answers to the BFI-2-S were analysed. Domain scale scores for the five personality traits were generated, as per the scoring key.

### 2.6. Statistical analysis

The data were summarised using descriptive statistics, by wave. Where possible, representativeness of the sample was assessed in comparison to publicly available data (41).

We were interested in understanding the progression of student wellbeing over the time period of the study. As such, for those who responded to three or more of the study waves, a series of latent profile analyses (29) (sometimes referred to as mixture modelling) were performed to identify latent subpopulations of students, using WEMWBS scores as continuous indicators, over the five waves of the study. This latent trajectory model was not a growth model. Rather, it was a longitudinal latent class model (42). Given that the observed indicators were continuous rather than categorical, in this case it is referred to as a longitudinal latent profile model. This model used full information maximum likelihood to estimate the presence of postulated latent categorical factors that explained the correlation between measures at different timepoints with observations (in this case, participants) (43). The optimal number of latent profiles was identified using the Bayesian information criterion (BIC), with smaller values indicating better model fit (44). We initially started with a one-class model, and incrementally increased the number of classes evaluated until optimal fit was identified. Model fit statistics for all models considered are available in Additional File 1.

Our latent classes were estimated on a subpopulation of our sample. In order to understand how representative this subpopulation was, logistic regression models were used to assess the response propensity of participants to respond to three or more waves of the survey (in comparison to one or two waves), using demographic, educational and BFI-2-S variables. Multinomial logit models were then used to model the relationship between the available demographic, educational, mental health and personality variables, and the identified latent classes.

Descriptive statistics and logit models were performed in Stata version 17 (45), and latent profile analyses were performed in Mplus version 8 (46).

## 3. Results

### 3.1. Descriptive statistics

In total, data were collected on 4,622 students who participated in at least one wave of the survey. A summary of the cohort statistics is displayed in Table 1. Across W1 to W4, the survey populations were relatively consistent across demographic and educational variables. However, respondents to W5 of the survey were somewhat different; compared to the earlier waves, W5 students comprised of a lower proportion of home students and those who self-reported White ethnicity. As can be seen, of those who responded, across all waves a substantial majority identified as female.

**Table 1:** Demographics, educational and mental health data and response rates in each wave of the survey.

	Wave 1 (September/October 2019)		Wave 2 (March/April 2020)		Wave 3 (September/October 2020)		Wave 4 (March/April 2021)		Wave 5 (September/October 2021)	
Participants	*n* = 1503		*n* = 1250		*n* = 1505		*n* = 1293		*n* = 1948	
	*n* (%)	Missing (%)	*n* (%)	Missing (%)	*n* (%)	Missing (%)	*n* (%)	Missing (%)	*n* (%)	Missing (%)
Male	388/1452 (26.7%)	51 (3.39%)	284/1223 (23.2%)	27 (2.16%)	374/1455 (25.7%)	50 (3.32%)	341/1249 (27.3%)	44 (3.40%)	583/1876 (31.8%)	72 (3.70%)
Non-white ethnicity	196/1475 (13.3%)	28 (1.86%)	184/1236 (14.9%)	14 (1.12%)	224/1484 (15.1%)	21 (1.40%)	187/1275 (14.7%)	18 (1.39%)	584/1899 (30.8%)	49 (2.52%)
LGBT+ sexuality	463/1415 (32.7%)	88 (5.85%)	377/1187 (31.8%)	63 (5.04%)	466/1422 (32.8%)	83 (5.51%)	402/1212 (33.2%)	81 (6.26%)	566/1768 (32.0%)	180 (9.24%)
One or more disability	222/560 (39.6%)	943 (62.7%)	228/594 (38.4%)	656 (52.5%)	517/1442 (35.9%)	63 (4.19%)	379/1110 (34.1%)	183 (14.2%)	483/1771 (27.3%)	177 (9.09%)
Parents have higher education	403/557 (72.4%)	946 (62.9%)	430/591 (72.8%)	659 (52.7%)	1013/1445 (70.1%)	60 (3.99%)	790/1108 (71.3%)	185 (14.3%)	1238/1795 (69.0%)	153 (7.85%)
Postgraduate student	221/798 (27.7%)	705 (46.9%)	341/1199 (28.4%)	51 (4.08%)	379/1321 (28.7%)	184 (12.2%)	373/1157 (32.2%)	136 (10.5%)	636/1845 (34.5%)	103 (5.29%)
Overseas student	258/1486 (17.4%)	17 (1.13%)	228/1245 (18.3%)	5 (0.40%)	273/1494 (18.3%)	11 (0.73%)	245/1284 (19.1%)	9 (0.70%)	644/1917 (33.6%)	31 (1.59%)
Faculty	-	681 (45.3%)	-	23 (1.84%)	-	158 (10.5%)	-	114 (8.82%)	-	58 (2.98%)
Arts and Humanities	207/822 (25.2%)	-	310/1227 (25.3%)	-	330/1347 (24.5%)	-	301/1179 (25.5%)	-	431/1890 (22.8%)	-
Sciences	407/822 (49.5%)	-	577/1227 (47.0%)	-	659/1347 (48.9%)	-	591/1179 (50.1%)	-	820/1890 (43.4%)	-
Social Sciences	208/822 (25.3%)	-	340/1227 (27.7%)	-	358/1347 (26.6%)	-	287/1179 (24.3%)	-	639/1890 (33.8%)	-
Previously sought help for a mental health condition	714/1460 (48.9%)	43 (2.86%)	579/1226 (47.2%)	24 (1.92%)	637/1469 (43.4%)	36 (2.39%)	565/1259 (44.9%)	34 (2.63%)	697/1851 (37.7%)	97 (4.98%)
Previously diagnosed with a mental health condition	486/1449 (33.5%)	54 (3.59%)	383/1213 (31.6%)	37 (2.96%)	410/1453 (28.2%)	52 (3.46%)	373/1255 (29.7%)	38 (2.94%)	445/1832 (24.3%)	116 (5.95%)
Index of multiple deprivation (Mean, SD)	7.02 (2.58)	361/1503 (24.0%)	7.04 (2.57)	284/1250 (22.7%)	7.03 (2.58)	354/1505 (23.5%)	6.95 (2.58)	327/1293 (25.3%)	7.07 (2.62)	573/1948 (29.4%)
	**Mean (SD)**		**Mean (SD)**		**Mean (SD)**		**Mean (SD)**		**Mean (SD)**	
WEMWBS	43.82 (9.79)		41.53 (9.95)		42.52 (9.65)		40.36 (9.82)		44.16 (10.39)	
GP-CORE	25.28 (8.86)		26.18 (9.85)		24.56 (9.54)		26.24 (9.76)		24.15 (8.70)	
PHQ-8 (*n* = 1,276)	n/a		n/a		n/a		10.11 (5.65)		n/a	
GAD-7 (*n* = 1,276)	n/a		n/a		n/a		8.62 (5.34)		n/a	
ULS-8 (*n* = 1,264)	n/a		n/a		n/a		20.56 (2.12)		n/a	
BFI-2-S: agreeableness (*n* = 2,365)	22.62 (4.18)									
BFI-2-S: conscientiousness (*n* = 2,367)	20.40 (4.90)									
BFI-2-S: extraversion (*n* = 2,366)	17.64 (5.12)									
BFI-2-S: negative emotions (*n* = 2,366)	20.14 (5.74)									
BFI-2-S: open mindedness (*n* = 2,364)	22.24 (4.30)									

BFI-2-S, Big Five Inventory-2 short form; GAD-7, Generalised Anxiety Disorder-7; GP-CORE, General Population Clinical Outcomes in Routine Evaluation; LGBT+, Lesbian, Gay, Bisexual, Transgender, or other sexual identities; PHQ-8, Patient Health Questionnaire-8; SD, Standard deviation; ULS-8, UCLA Loneliness Scale; WEMWBS, Warwick-Edinburgh Mental Wellbeing Scale.

The University routinely publishes public data relating to the total number of students enrolled, and some demographics (41). In 2019/2020 (i.e., W1 to W4), a total of 18,930 students were enrolled at the University: of which 13,690 (72.3%) were undergraduate students; 8,225 (43.5%) were male; 5,225 (27.6%) were non-White ethnicity; and 2,970 (15.7%) reported a disability. In 2020/2021 (W5), a total of 20,435 students were enrolled: 14,560 (71.2%) were undergraduate students; 8,945 (43.8%) were male; 6,040 (30.0%) were non-White ethnicity; and 3,365 (16.5%) reported a disability. Data on sexuality are not routinely reported. Therefore, response rates across the whole student population varied between 6.6% (W2) and 9.5% (W5). Other than in W1 (where substantial missing data were recorded), undergraduate students were somewhat underrepresented relative to the University population as a whole across our survey. Males were underrepresented across all waves of our survey, as were those reporting non-White ethnicity, with the exception of W5 where the sample appeared representative of the population. Around 30–40% of those who provided data in our survey reported one or more disabilities, in comparison to around 16% of the wider student population. However, it is unclear whether the University wide measure included mental health conditions, as our measure did. Furthermore, response rates for this measure in our survey were low, particularly in the first two waves.

Mean WEMWBS scores for each subpopulation at each wave appeared broadly similar, as did mean GP-CORE scores and the proportion of respondents who indicated they had been diagnosed with a mental health condition. The proportion of those reporting previously having sought help for a mental health condition was also reasonably consistent, although did reduce somewhat in W5.

Response rates to individual questions was generally high across most of the waves. Substantial missing data were observed for the Index of Multiple Deprivation, and also in W1 and W2 for a number of items (disability, parental education and level of study). For the latter items, response rates were higher from W3 onwards.

### 3.2. Response propensity

The majority of participants only responded to one wave of the five (*n* = 2,904, 62.8%), with decreasing numbers responding to two waves (*n* = 953, 20.6%), three waves (*n* = 456, 9.87%) and four waves (*n* = 224, 4.85%). A small number of students responded to all five waves (*n* = 85, 1.84%). Around 15% of the total participants responded to three or more waves of the survey (*n* = 765, 16.6%).

Table 2 displays the univariable odds ratios (ORs) when modelling whether someone responded to three or more waves of the survey. Frequent responders were less likely to be male (OR 0.75, 0.63 to 0.91, *p* = 0.03), report non-White ethnicity (OR 0.42, 0.34 to 0.53, *p* < 0.001), be a postgraduate student (OR 0.79, 0.66 to 0.95, *p* = 0.01), be an overseas student (OR 0.51, 0.41 to 0.62, *p* < 0.001), or to be based in the Faculty of Social Sciences. Frequent responders were also more likely to be disabled (OR 1.57, 1.31 to 1.87, *p* < 0.001), have a parent with higher education (OR 1.35, 1.11 to 1.63, *p* = 0.002), and have higher scores on the conscientiousness domain of the BFI-2-S (OR 1.03, 1.01 to 1.05, *p* = 0.001).

**Table 2:** Univariable and multivariable odds ratios for modelling three responses or more to the survey.

Variable	Univariable OR (95% CI) *p*-value	Multivariable OR (95% CI) *p*-value
Male	0.75 (0.63 to 0.91) *p* = 0.03	0.80 (0.62 to 1.02) *p* = 0.08
Non-White ethnicity	0.42 (0.34 to 0.53) *p* < 0.001	0.64 (0.45 to 0.90) *p* = 0.01
LGBT+ sexuality	1.18 (1.00 to 1.40) *p* = 0.05	n/a
Postgraduate student	0.79 (0.66 to 0.95) *p* = 0.01	1.05 (0.82 to 1.35) *p* = 0.70
Previously sought help for a mental health condition	1.13 (0.97 to 1.33) *p* = 0.12	n/a
Previously diagnosed with a mental health condition	1.16 (0.98 to 1.38) *p* = 0.09	n/a
Overseas student	0.51 (0.41 to 0.62) *p* < 0.001	0.68 (0.49 to 0.95) *p* = 0.02
Parents have higher education	1.35 (1.11 to 1.63) *p* = 0.002	1.41 (1.11 to 1.78) *p* = 0.004
One or more disability	1.57 (1.31 to 1.87) *p* < 0.001	1.23 (0.99 to 1.55) *p* = 0.07
Faculty		
Sciences (vs. Arts and Humanities)	1.13 (0.92 to 1.38) *p* = 0.22	0.98 (0.76 to 1.27) *p* = 0.91
Social Sciences (vs. Arts and Humanities)	0.63 (0.49 to 0.79) *p* < 0.001	0.75 (0.56 to 1.02) *p* = 0.07
Social Sciences (vs. Sciences)	0.55 (0.45 to 0.68) *p* < 0.001	0.77 (0.59 to 1.00) *p* = 0.05
BFI-2-S: agreeableness	1.00 (0.98 to 1.02) *p* = 0.91	n/a
BFI-2-S: conscientiousness	1.03 (1.01 to 1.05) *p* = 0.001	1.04 (1.02 to 1.07) *p* < 0.001
BFI-2-S: extraversion	1.00 (0.98 to 1.02) *p* = 0.93	n/a
BFI-2-S: negative emotions	1.01 (0.99 to 1.02) *p* = 0.39	n/a
BFI-2-S: open-mindedness	1.00 (0.98 to 1.02) *p* = 0.77	n/a

Only those variables statistically significant at the *p* = 0.05 level on univariable analysis were included in the multivariable model. BFI-2-S, Big Five Inventory-2 short form; LGBT+, Lesbian, Gay, Bisexual, Transgender, or other sexual identities.

In a multivariable model, including all variables which were statistically significant on univariable analysis, only non-White ethnicity (OR 0.64, 0.45 to 0.90, *p* = 0.01), being an overseas student (OR 0.68, 0.49 to 0.95, *p* = 0.02), having a parent with higher education (OR 1.41, 1.11 to 1.78, *p* = 0.004), and conscientiousness (OR 1.04, 1.02 to 1.07, *p* < 0.001) were statistically significant predictors of responding to three or more waves of the survey.

### 3.3. Longitudinal latent profile analysis

**Figure 2:**
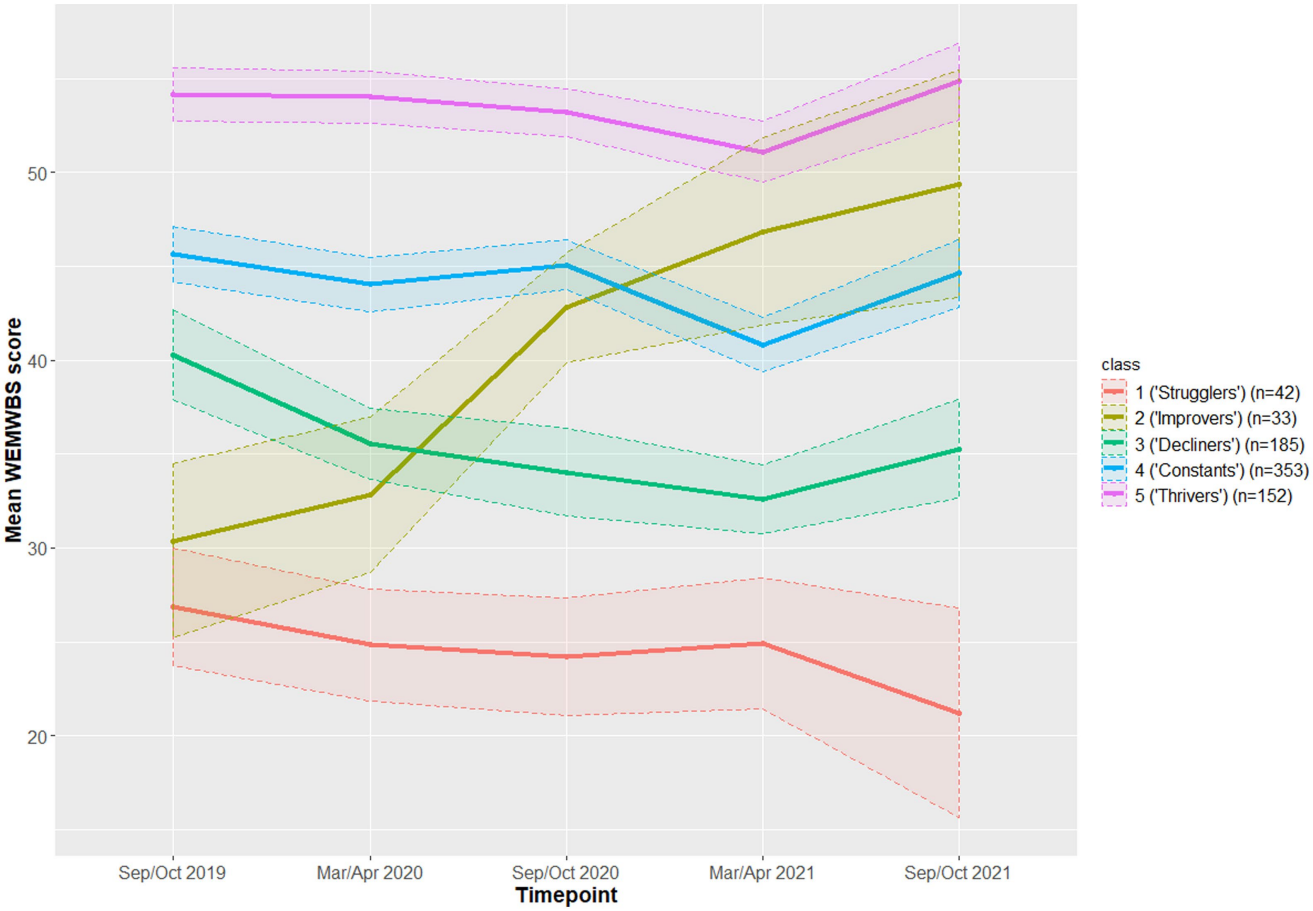
Latent trajectories, and associated 95% confidence intervals, for the five-class model.

Optimal model fit, as indicated by the smallest Bayesian Information Criterion (BIC) value, was observed for a five-class model (BIC = 19203.00) for WEMWBS. The latent profile trajectories, and associated 95% confidence intervals, are displayed in Figure 2 (full results are available in Additional File 1, alongside information criterion values for the other models considered). Around 20% of students consistently displayed relatively high WEMWBS scores (class 5, *n* = 152, WEMWBS > 51.1), indicating positive levels of wellbeing. A small number of students (class 1 and class 2) started the study period with low WEMWBS scores (26.9 and 30.4). Two trajectories (classes 4 and 5) were broadly stable across the period of the study, though both showed a reduction in reported wellbeing at one wave. These classes were the two with the highest initial, pre-pandemic scores (and therefore higher wellbeing) and comprised two thirds of students (total *n* = 505/765, 66%). Two classes (3 and 1) showed a deterioration in scores across time, though with broad confidence intervals for at least one of the groups. These two classes represented 30% of students (total *n* = 227/765). A final small class (class 2) started with low scores but showed substantial improvement in WEMWBS scores over the study, rising from 30.4 (26.0 to 34.7) at wave 1, to 49.4 (43.7 to 55.1) at wave 5 (*n* = 33, 4%).

To facilitate interpretation of the findings we gave the following descriptive labels to the latent profile trajectories identified as follows:

- Class 1: ‘Strugglers’—students reporting a low level of wellbeing which declined further at W5.
- Class 2: ‘Improvers’—students reporting an initial low level of wellbeing which improved over the course of the study.
- Class 3: ‘Decliners’—those showing approximately average levels of wellbeing with some evidence of decline over the course of the study.
- Class 4: ‘Constants’—those reporting above average wellbeing, with only a modest dip in the period immediately following the pandemic.
- Class 5: ‘Thrivers’—students reporting high levels of wellbeing, increasing further at W5.

### 3.4. Identification of class membership

Owing to the small number of identified individuals in class 1 and class 2 (‘strugglers’ and ‘improvers’, respectively), we proceeded only with univariable multinomial logistic regression. As the multinomial logit model relies on references between two outcome classes, we present only certain pairwise comparisons here. Full results are available in Additional File 1.

#### 3.4.1. Class 1 (‘strugglers’) vs. class 2 (‘improvers’)

These two classes started with low WEMWBS scores. However, over the period of the study, the ‘strugglers’ displayed worsening wellbeing, whereas ‘improvers’ showed substantial increases. Significant univariable predictors of being a ‘struggler’ (class 1) instead of an ‘improver’ (class 2) were previously being diagnosed with a mental health condition [Relative Risk Ratio (RRR) 2.72, 1.03 to 7.26, *p* = 0.04] and being disabled (RRR 5.05, 1.42 to 17.92, *p* = 0.01). The interpretation of the first result is as follows: those who reported previously being diagnosed with a mental health condition had nearly three times the risk ratio of being in the ‘struggler’ class, compared to the ‘improver’ class. Those who scored higher on agreeableness were also less likely to be ‘strugglers’ compared to ‘improvers’ (RRR 0.83, 0.73 to 0.94, *p* = 0.004). Those who scored themselves higher on negative emotion were more likely to be ‘strugglers’ compared to ‘improvers’ (RRR 1.29, 1.12 to 1.49, *p* = 0.001).

#### 3.4.2. Class 1 (‘strugglers’) vs. class 3 (‘decliners’)

These two classes displayed similar wellbeing trajectories throughout the study period, with both showing reduced WEMWBS scores over time. The major difference between these two classes was that ‘decliners’ had higher wellbeing scores than ‘strugglers’. Those with higher agreeableness scores were more likely to be ‘decliners’ than ‘strugglers’ (RRR 1.14, 1.04 to 1.23, *p* = 0.003). In contrast, those with higher negative emotion scores (RRR 0.84, 0.74 to 0.95, *p* = 0.004), and those previously diagnosed with (RRR 0.21, 0.10 to 0.46, *p* < 0.001), or sought help for (RRR 0.27, 0.12 to 0.63, *p* = 0.002), a mental health condition, were less likely to be ‘decliners’ than ‘strugglers’.

#### 3.4.3. Class 2 (‘improvers’) vs. class 3 (‘decliners’)

At the start of the study, ‘decliners’ had higher wellbeing scores than ‘improvers’, although both were low. However, whilst ‘improvers’ displayed much higher wellbeing by the end of the study, the wellbeing scores of ‘decliners’ had reduced somewhat. Only one variable was a statistically significant predictor for this comparison, with overseas students more likely to be ‘improvers’ than ‘decliners’ (RRR 2.78, 1.15 to 6.74, *p* = 0.02).

#### 3.4.4. Class 2 (‘improvers’) vs. class 5 (‘thrivers’)

‘Improvers’ started the study with much lower WEMWBS scores than ‘thrivers’, but by the end of the study these two classes had comparable WEMWBS scores. Significant predictors of being a ‘thriver’ rather than an ‘improver’ were being male (RRR 3.69, 1.07 to 12.8, *p* = 0.04), and higher scores on conscientiousness (RRR 1.15, 1.05 to 1.26, *p* = 0.002), extraversion (RRR 1.20, 1.10 to 1.31, *p* < 0.001) and open-mindedness (RRR 1.14, 1.03 to 1.25, *p* = 0.01). In contrast, LGBT+ students (RRR 0.26, 0.12 to 0.56, *p* = 0.001), those reporting at least one disability (RRR 0.17, 0.07 to 0.38, *p* < 0.001), and those who have previously sought help for (RRR 0.31, 0.14 to 0.67, *p* = 0.003), or been diagnosed with (RRR 0.19, 0.08 to 0.44, *p* < 0.001) a mental health condition, were less likely to be ‘thrivers’ than ‘improvers’.

#### 3.4.5. Class 4 (‘constants’) vs. class 5 (‘thrivers’)

Significant predictors of being a ‘thriver’ rather than a ‘constant’ were higher scores on extraversion (RRR 1.12, 1.07 to 1.17, *p* < 0.001), conscientiousness (RRR 1.11, 1.06 to 1.16, p < 0.001) and open mindedness (RRR 1.08, 1.03 to 1.13, *p* = 0.003). In contrast, LGBT+ students (RRR 0.57, 0.36 to 0.90, *p* = 0.02), those previously diagnosed with a mental health condition (RRR 0.51, 0.31 to 0.83, *p* = 0.01), those with a disability (RRR 0.60, 0.37 to 0.97, *p* = 0.04) and those scoring higher on negative emotion (RRR 0.86, 0.83 to 0.90, p < 0.001) were less likely to be a ‘thriver’ rather than a ‘constant’.

#### 3.4.6. Class 3 (‘decliners’) vs. class 5 (‘thrivers’)

These two classes had similar trajectories throughout the pandemic, with the main difference being baseline WEMWBS scores. There were a number of significant univariable predictors of being a ‘thriver’ rather than a ‘decliner’. Only overseas students (RRR 1.82, 1.00 to 3.32, *p* = 0.05) and those whose parents had participated in higher education (RRR 1.95, 1.13 to 3.32, *p* = 0.01) were more likely to be classed as ‘thrivers’ compared to ‘decliners’. In contrast, students identifying as LGBT+ (RRR 0.36, 0.26 to 0.58, *p* < 0.001), those who had previously sought help for mental health issues (RRR 0.41, 0.26 to 0.64, *p* < 0.001) or been diagnosed with a mental health condition (RRR 0.33, 0.19 to 0.56, *p* < 0.001), or those reporting a disability (RRR 0.22, 0.13 to 0.37, *p* < 0.001) were less likely to be a ‘thriver’ compared to a ‘decliner’. Scores on all five domains of the BFI-2-S were statistically significant predictors. Scoring higher on agreeableness (RRR 1.11, 1.05 to 1.18, *p* = 0.001), conscientiousness (RRR 1.25, 1.19 to 1.33, *p* < 0.001) extraversion (RRR 1.23, 1.17 to 1.29, *p* < 0.001) and open-mindedness (RRR 1.09, 1.03 to 1.15, *p* = 0.002) were associated with higher relative risk ratios of being in the ‘thrivers’ rather than ‘decliners’ class. In contrast, those with higher negative emotion scores were less likely to be a ‘thriver’ compared to a ‘decliner’ (RRR 0.70, 0.65 to 0.74, *p* < 0.001).

## 4. Discussion

This paper reports the first findings from the Student Wellbeing at Northern England Universities (SWANS) study in which we identified five latent classes of student wellbeing, measured by WEMWBS, over the period of study. Two classes (‘constants’ and ‘thrivers’), making up two thirds of the sample, showed a broadly stable trajectory for mental wellbeing. These students reported higher levels of wellbeing relative to the other classes before the onset of the pandemic. Two groups (‘strugglers’ and ‘decliners’), making up around 30% of the sample, generally reported at least modest reductions in wellbeing. Finally, one class (‘improvers’), small in size (*n* = 33, 4%), showed a substantial and sustained improvement in wellbeing across the measurement points.

In general, the latent trajectories of wellbeing we identified were relatively stable over the study period, with one major exception (the ‘improver’ class). This would imply that psychological wellbeing throughout the period of the pandemic, and indeed throughout university studies in general, are largely determined by baseline and previous levels of wellbeing. Previous studies that also used a longitudinal design reported a broadly negative impact of the pandemic on symptoms in students (25–28), though only one study examined longer-term effects and this found some improvement in symptoms after the end of lockdown (27). This study complements the existing literature in identifying different trajectories of change for different groups, allowing for a more nuanced view of changes in wellbeing.

The main predictors of membership of a worse wellbeing trajectory appeared to be reporting a disability or previous mental health diagnosis. Some sociodemographic variables were associated with class membership, with those factors traditionally underrepresented in higher education generally associated with less favourable wellbeing trajectories. These findings are broadly in line with previous evidence of risk factors in UK university students (17). It has been well reported that the COVID-19 pandemic disproportionately impacted on traditionally disadvantaged communities, not only in terms of the direct impact of health risks (47), but also economically and socially (48, 49). Students from such backgrounds were thus likely to be disproportionately impacted by the pandemic, which may have had additional impacts on the wellbeing measures assessed in this study.

A lack of a previous mental health diagnosis and higher levels of reported agreeableness were associated with being in the ‘improvers’ class compared to those whose wellbeing stayed very low throughout the study period. In this respect, students with high levels of prosocial traits may have been more effective at increasing social connectedness, and thus mitigating against their initial, pre-pandemic, low levels of reported wellbeing. Nevertheless, it must be emphasised that this class represented a small minority of the overall sample. Thus, most students in our sample report a modest impact of the pandemic on their wellbeing, if any. Certainly, for a generation who have grown up with the internet and mobile technology even the relative geographical isolation of the pandemic and associated lock-downs will not have prevented remote social contact in most cases. Moreover, despite the disruption to face-to-face teaching brought by social distancing, most higher education institutions quickly adapted, providing numerous remote, digital learning opportunities (10).

### 4.1. Strengths and limitations

This study had a number of strengths and limitations. We collected a wide range of data on student wellbeing and mental health over an extended time period, which provided an opportunity to examine the longitudinal impact of the pandemic on student wellbeing. Students were recruited before the start of the pandemic, throughout its course, including a series of lockdowns and other restrictions on permitted activity, and to the end of those restrictions and a return to relative normality. This provided crucial information on how students wellbeing changed throughout this period. The use of latent profile analysis enabled us to identify groups of individuals similar in terms of their response to the COVID-19 pandemic and the associated societal changes. That being said, caution is needed in causally attributing change in wellbeing to the pandemic. It is possible, though unlikely, in principle, that similar trajectories would have been observed in the absence of the pandemic. Data collection was independent of the university and anonymous, potentially reducing response biases in groups less likely to declare mental health issues.

However, despite the survey being designed to be low-burden, participation rates were low, and comparison with data available on the wider student population at the University indicates that the sample may be unrepresentative of the population in a number of ways (e.g., undergraduates, males, and those reporting non-White ethnicity were underrepresented). Furthermore, there is some indication that those students who completed the questionnaires across three or more waves differed from those who completed it fewer times. It is unclear what affect this has on the conclusions drawn about different trajectories. Owing to small numbers in two classes we were unable to estimate multivariable models when predicting class membership. Thus, we were not in a position to infer whether the associations with demographic and personality factors were independent of each other. We dichotimised data relating to gender identity and ethnicity in this study. This was done for analytic purposes. However, this has limitations with regards to the conclusions we can draw from these groups.

### 4.2. Potential implications for practice

In line with other studies, we identified a minority of around 10% of our sample who reported consistently low levels of wellbeing. These students were generally more likely to identify as part of minority groups and have a previous history of mental health difficulties. Moreover, if anything, their wellbeing declined after the pandemic. The benefits of promoting wellbeing are widely recognised, to the extent that it is one of the United Nation’s Sustainable Development Goals for 2030 (50). Such issues also have an impact on educational outcomes (17), which will likely have implications for future careers and life courses. Universities offer a range of resources for wellbeing support, such as self-help apps, guided mindfulness and financial support for student clubs. However, whilst there is some evidence for at least some short-term effectiveness of such interventions, it remains an under researched area in student populations (51). It may be that screening processes, either at the point of application, or matriculation would provide opportunities for targeted support or signposting for students at high risk of consistently poor mental health and wellbeing. There is also a clear opportunity to explore the characteristics of students at risk of poor mental health during their studies, and, more importantly, what identification processes and interventions are likely to be both clinically and cost-effective. It may be that there are also opportunities to evaluate new models of service delivery, with increased levels of collaboration and integration between support services delivered by universities and healthcare providers. The degree to which such resources are a matter for higher education institutions vs. health services to provide is a matter for debate and discussion. However, the reality is that, in the UK and elsewhere, demand for mental health services was far outstripping demand, even prior to the pandemic (52).

The intriguing finding that a small number of students with initially low levels of wellbeing showed marked improvements is worthy of further exploration. It is possible that for some students working remotely away from the university was beneficial, at least in the short-term. There may be value in qualitative work to explore whether there is anything universities can learn from this to support students who may find the demands of life at university a struggle. Future work should also investigate differences across different ethnic minority groups, and those who identify as non-binary. Additionally, our relatively low participation rates indicate the importance of work to increase and maintain student engagement with longitudinal research. There is scope, therefore, for future research to explore ways of increasing engagement within this population. Indeed, work is underway by the Student Mental Health Research Network to provide guidance on the setup and running of student longitudinal wellbeing studies following recommendations from cross-sector consensus groups (53).

### 4.3. Conclusion

Our findings suggest that in the event of further pandemics, or other external stressors, universities may benefit from targeting help at those with initially lower levels of wellbeing. In particular, those students who are already struggling may be especially adversely affected without additional support. Moreover, in the absence of acute external pressures our results indicate that there are a minority of students who may benefit from proactive targeted support. These may be those from less advantaged socioeconomic backgrounds and minority groups, such as those identifying as LGBT+. Drop out or under achievement at higher education is associated with high personal and societal costs. Thus, investing in further research and targeted support for those at risk of poor educational outcomes secondary to mental health issues could be cost effective and merits additional exploration.

## Data availability statement

The datasets presented in this article are not readily available because participants did not provide consent to share data. The code used for data analysis are available on request. Requests to access the code should be directed to LP, lewis.paton@york.ac.uk.

## Ethics statement

The Department of Health Sciences Research Governance Committee, University of York provided ethical approval for the study (HSRGC/2019/346/J). Written informed consent for participation was obtained on completion of the study survey.

## Author contributions

LP, PT, MB, BB, EB, LE, LK, DM, and PH contributed to the conception of the study, interpretation of data, and drafting the paper. PH led on data collection. LP led on data analysis, with support from PT. All authors contributed to the article and approved the submitted version.

## Funding

The set-up of the cohort was funded by alumni donations at the University of York. A portion of PH’s time on the project was funded by the Student Mental Health Research Network (SMaRteN), one of eight UK Research and Innovation funded mental health research networks, grant number ES/S00324X/1.

## Conflict of interest

The authors declare that the research was conducted in the absence of any commercial or financial relationships that could be construed as a potential conflict of interest.

## Publisher’s note

All claims expressed in this article are solely those of the authors and do not necessarily represent those of their affiliated organizations, or those of the publisher, the editors and the reviewers. Any product that may be evaluated in this article, or claim that may be made by its manufacturer, is not guaranteed or endorsed by the publisher.

## Abbreviations

BIC: Bayesian Information Criterion
BFI-2-S: Big Five Inventory-2 short form
GAD-7: Generalised Anxiety Disorder-7
GP-CORE: General Population Clinical Outcomes in Routine Evaluation
LGBT+: Lesbian, Gay, Bisexual, Transgender, or other sexual identities
OR: Odds ratio
PHQ-8: Patient Health Questionnaire-8
RRR: Relative risk ratio
SD: Standard deviation
SWANS: Student Wellbeing at Northern England Universities
ULS-8: UCLA loneliness scale
W1, …, W5: Wave 1, …, Wave 5
WEMWBS: Warwick-Edinburgh Mental Wellbeing Scale
