# Supplementary material for: Mental health trajectories in university students across the COVID-19 pandemic: findings from the Student Wellbeing at Northern England Universities prospective cohort study
Source: Front Public Health. 2023 Jul 17;11:1188690. doi: 10.3389/fpubh.2023.1188690 (PMC10387533; doi:10.3389/fpubh.2023.1188690)
Supplement: Supplementary file 1 [file Table_1.docx]

**Additional file for Mental Health Trajectories in University Students Across the Covid-19 Pandemic: Findings from the Student Wellbeing At Northern England Universities** **(SWANS) Longitudinal Cohort Study**

Lewis W Paton, Paul A Tiffin, Michael Barkham, Bridgette M Bewick, Emma Broglia, Lisa Edwards, Louise Knowles, Dean McMillan, Paul N Heron

| **Class** | **Mean WEMWBS**  **(95% CI)** | | | | |
| --- | --- | --- | --- | --- | --- |
|  | **Wave 1** | **Wave 2** | **Wave 3** | **Wave 4** | **Wave 5** |
| **1 (‘Strugglers’)**  **n=42**  **(5.49%)** | 26.9  (21.8 to 31.9) | 24.8  (20.0 to 29.7) | 24.2  (17.8 to 30.6) | 24.9  (18.5 to 31.4) | 21.2  (12.6 to 29.8) |
| **2 (‘Improvers’)**  **n=33**  **(4.31%)** | 30.4  (26.0 to 34.7) | 32.9  (25.5 to 40.2) | 42.8  (39.3 to 46.3) | 46.8  (39.6 to 54.1) | 49.4  (43.7 to 55.1) |
| **3 (‘Decliners’)**  **n=185**  **(24.2%)** | 40.3  (35.3 to 45.3) | 35.5  (31.8 to 39.3) | 34.0  (29.3 to 38.8) | 32.6  (29.8 to 35.4) | 35.3  (30.6 to 39.9) |
| **4 (‘Constants’)**  **n=353**  **(46.1%)** | 45.6  (43.1 to 48.2) | 44.1  (41.3 to 46.9) | 45.1  (42.7 to 47.5) | 40.8  (38.5 to 43.2) | 44.6  (42.0 to 47.2) |
| **5 (‘Thrivers’)**  **n=152**  **(19.9%)** | 54.2  (52.6 to 55.8) | 54.0  (52.3 to 55.8) | 53.2  (51.8 to 54.6) | 51.1  (49.1 to 53.1) | 54.8  (52.5 to 57.2) |

**Table S1:** Mean WEMWBS score, and associated 95% confidence interval, across the five waves of the survey for each of the five identified latent classes. WEMWBS: Warwick-Edinburgh Mental Wellbeing scale

| **Variable** | **Class 1**  **(‘Strugglers’)** | **Class 2**  **(‘Improvers’)** | **Class 3**  **(‘Decliners’)** | **Class 4**  **(‘Constants’)** | **Class 5**  **(‘Thrivers’)** |
| --- | --- | --- | --- | --- | --- |
| **Male** | REF | 0.30  (0.07 to 1.22)  p=0.09 | 1.01  (0.44 to 2.30)  p=0.99 | 0.91  (0.41 to 2.01)  p=0.81 | 1.11  (0.48 to 2.55)  p=0.81 |
| **Non-white ethnicity** | REF | 1.60  (0.51 to 4.99)  p=0.42 | 0.83  (0.33 to 2.06)  p=0.69 | 0.47  (0.19 to 1.14)  p=0.09 | 0.77  (0.30 to 1.97)  p=0.58 |
| **LGBT+ sexuality** | REF | 1.06  (0.42 to 2.67)  p=0.90 | 0.77  (0.38 to 1.52)  p=0.45 | 0.48  (0.25 to 0.93)  p=0.03 | 0.27  (0.13 to 0.57)  p=0.001 |
| **Postgraduate student** | REF | 0.94  (0.35 to 2.49)  p=0.89 | 0.55  (0.26 to 1.17)  p=0.12 | 0.70  (0.35 to 1.42)  p=0.33 | 0.76  (0.36 to 1.61)  p=0.48 |
| **Previously sought help for a mental health condition** | REF | 0.36  (0.13 to 1.02)  p=0.06 | 0.27  (0.12 to 0.63)  p=0.002 | 0.16  (0.07 to 0.36)  p<0.001 | 0.11  (0.05 to 0.26)  p<0.001 |
| **Previously diagnosed with a mental health condition** | REF | 0.37  (0.14 to 0.98)  p=0.04 | 0.21  (0.10 to 0.46)  p<0.001 | 0.14  (0.07 to 0.29)  p<0.001 | 0.07  (0.03 to 0.16)  p<0.001 |
| **Overseas student** | REF | 1.88  (0.61 to 5.72)  p=0.27 | 0.67  (0.27 to 1.70)  p=0.41 | 0.92  (0.39 to 2.18)  p=0.86 | 1.23  (0.50 to 3.04)  p=0.66 |
| **Parents have higher education** | REF | 0.85  (0.30 to 2.38)  p=0.75 | 0.84  (0.28 to 1.83)  p=0.66 | 1.41  (0.66 to 3.00)  p=0.37 | 1.63  (0.72 to 3.70)  p=0.24 |
| **One or more disability** | REF | 0.20  (0.06 to 0.70)  p=0.01 | 0.15  (0.05 to 0.45)  p=0.001 | 0.06  (0.02 to 0.16)  p<0.001 | 0.03  (0.01 to 0.10)  p<0.001 |
| **BFI-2S: Agreeableness** | REF | 1.20  (1.06 to 1.37)  p=0.004 | 1.14  (1.04 to 1.24)  p=0.003 | 1.21  (1.11 to 1.32)  p<0.001 | 1.26  (1.15 to 1.39)  p<0.001 |
| **BFI-2S: Conscientiousness** | REF | 1.10  (0.99 to 1.22)  p=0.08 | 1.01  (0.94 to 1.09)  p=0.75 | 1.15  (1.06 to 1.24)  p<0.001 | 1.27  (1.17 to 1.38)  p<0.001 |
| **BFI-2S: Extraversion** | REF | 1.04  (0.93 to 1.16)  p=0.52 | 1.01  (0.93 to 1.10)  p=0.77 | 1.11  (1.03 to 1.20)  p=0.01 | 1.24  (1.15 to 1.35)  p<0.001 |
| **BFI-2S: Negative emotions** | REF | 0.78  (0.67 to 0.90)  p=0.001 | 0.84  (0.74 to 0.95)  p=0.004 | 0.68  (0.60 to 0.77)  p<0.001 | 0.58  (0.51 to 0.66)  p<0.001 |
| **BFI-2S: Open mindedness** | REF | 0.92  (0.82 to 1.04)  p=0.18 | 0.96  (0.89 to 1.05)  p=0.41 | 0.98  (0.90 to 1.06)  p=0.55 | 1.05  (0.96 to 1.15)  p=0.27 |
| **Index of multiple deprivation** | REF | 1.06  (0.87 to 1.31)  p=0.55 | 1.07  (0.93 to 1.24)  p=0.33 | 1.17  (1.02 to 1.34)  p=0.03 | 1.11  (0.96 to 1.29)  p=0.16 |

**Table S2:** Results from univariable multinomial logistic regression models, with class 1 as the reference category. BFI-2-S: Big Five Inventory-2 short form. LGBT+: Lesbian, Gay, Bisexual, Transgender, other.

| **Variable** | **Class 1**  **(‘Strugglers’)** | **Class 2**  **(‘Improvers’)** | **Class 3**  **(‘Decliners’)** | **Class 4**  **(‘Constants’)** | **Class 5**  **(‘Thrivers’)** |
| --- | --- | --- | --- | --- | --- |
| **Male** | 3.33  (0.82 to 13.6)  p=0.09 | REF | 3.36  (0.98 to 11.53)  p=0.05 | 3.02  (0.90 to 10.2)  p=0.07 | 3.69  (1.07 to 12.8)  p=0.04 |
| **Non-white ethnicity** | 0.63  (0.20 to 1.94)  p=0.42 | REF | 0.52  (0.21 to 1.27)  p=0.15 | 0.29  (0.12 to 0.70)  p=0.01 | 0.48  (0.19 to 1.21)  p=0.12 |
| **LGBT+ sexuality** | 0.94  (0.37 to 2.37)  p=0.90 | REF | 0.72  (0.34 to 1.52)  p=0.39 | 0.45  (0.22 to 0.92)  p=0.03 | 0.26  (0.12 to 0.56)  p=0.001 |
| **Postgraduate student** | 1.07  (0.40 to 2.85)  p=0.89 | REF | 0.59  (0.26 to 1.33)  p=0.21 | 0.75  (0.35 to 1.63)  p=0.47 | 0.82  (0.36 to 1.84)  p=0.62 |
| **Previously sought help for a mental health condition** | 2.76  (0.98 to 7.81)  p=0.06 | REF | 0.76  (0.36 to 1.62)  p=0.47 | 0.45  (0.22 to 0.93)  p=0.03 | 0.31  (0.14 to 0.67)  p=0.003 |
| **Previously diagnosed with a mental health condition** | 2.72  (1.03 to 7.26)  p=0.04 | REF | 0.58  (0.27 to 1.24)  p=0.16 | 0.38  (0.18 to 0.79)  p=0.01 | 0.19  (0.08 to 0.44)  p<0.001 |
| **Overseas student** | 0.53  (0.17 to 1.63)  p=0.27 | REF | 0.36  (0.14 to 0.87)  p=0.02 | 0.49  (0.22 to 1.16)  p=0.09 | 0.66  (0.28 to 1.56)  p=0.34 |
| **Parents have higher education** | 1.18  (0.42 to 3.33)  p=0.75 | REF | 0.99  (0.43 to 2.27)  p=0.98 | 1.67  (0.75 to 3.73)  p=0.21 | 1.93  (0.81 to 4.58)  p=0.14 |
| **One or more disability** | 5.05  (1.42 to 17.9)  p=0.01 | REF | 0.77  (0.35 to 1.68)  p=0.51 | 0.28  (0.13 to 0.60)  p=0.001 | 0.17  (0.07 to 0.38)  p<0.001 |
| **BFI-2S: Agreeableness** | 0.83  (0.73 to 0.94)  p=0.004 | REF | 0.95  (0.85 to 1.05)  p=0.29 | 1.01  (0.91 to 1.11)  p=0.91 | 1.05  (0.94 to 1.17)  p=0.37 |
| **BFI-2S: Conscientiousness** | 0.91  (0.82 to 1.01)  p=0.08 | REF | 0.92  (0.84 to 1.00)  p=0.06 | 1.04  (0.96 to 1.13)  p=0.35 | 1.15  (1.05 to 1.26)  p=0.002 |
| **BFI-2S: Extraversion** | 0.96  (0.87 to 1.08)  p=0.52 | REF | 0.98  (0.89 to 1.07)  p=0.60 | 1.07  (0.98 to 1.17)  p=0.11 | 1.20  (1.10 to 1.31)  p<0.001 |
| **BFI-2S: Negative emotions** | 1.29  (1.12 to 1.49)  p=0.001 | REF | 1.08  (0.98 to 1.19)  p=0.10 | 0.87  (0.80 to 0.95)  p=0.003 | 0.75  (0.68 to 0.83)  p<0.001 |
| **BFI-2S: Open mindedness** | 1.08  (0.96 to 1.22)  p=0.18 | REF | 1.04  (0.95 to 1.15)  p=0.37 | 1.06  (0.96 to 1.16)  p=0.25 | 1.14  (1.03 to 1.25)  p=0.01 |
| **Index of multiple deprivation** | 0.94  (0.77 to 1.15)  p=0.55 | REF | 1.01  (0.85 to 1.20)  p=0.92 | 1.10  (0.93 to 1.30)  p=0.26 | 1.05  (0.88 to 1.25)  p=0.62 |

**Table S3:** Results from univariable multinomial logistic regression models, with class 2 as the reference category. BFI-2-S: Big Five Inventory-2 short form. LGBT+: Lesbian, Gay, Bisexual, Transgender, other.

| **Variable** | **Class 1**  **(‘Strugglers’)** | **Class 2**  **(‘Improvers’)** | **Class 3**  **(‘Decliners’)** | **Class 4**  **(‘Constants’)** | **Class 5**  **(‘Thrivers’)** |
| --- | --- | --- | --- | --- | --- |
| **Male** | 0.99  (0.43 to 2.27)  p=0.99 | 0.30  (0.09 to 1.02)  p=0.05 | REF | 0.90  (0.59 to 1.37)  p=0.62 | 1.10  (0.67 to 1.80)  p=0.71 |
| **Non-white ethnicity** | 1.21  (0.49 to 3.00)  p=0.69 | 1.93  (0.79 to 4.74)  p=0.15 | REF | 0.56  (0.32 to 0.98)  p=0.04 | 0.93  (0.50 to 1.74)  p=0.82 |
| **LGBT+ sexuality** | 1.31  (0.66 to 2.60)  p=0.45 | 1.39  (0.66 to 2.93)  p=0.39 | REF | 0.62  (0.43 to 0.91)  p=0.02 | 0.36  (0.26 to 0.58)  p<0.001 |
| **Postgraduate student** | 1.81  (0.86 to 3.82)  p=0.12 | 1.69  (0.75 to 3.82)  p=0.21 | REF | 1.28  (0.83 to 1.97)  p=0.27 | 1.38  (0.83 to 2.30)  p=0.22 |
| **Previously sought help for a mental health condition** | 3.64  (1.60 to 8.30)  p=0.002 | 1.32  (0.62 to 2.81)  p=0.47 | REF | 0.59  (0.41 to 0.85)  p=0.01 | 0.41  (0.26 to 0.64)  p<0.001 |
| **Previously diagnosed with a mental health condition** | 4.67  (2.20 to 9.93)  p<0.001 | 1.71  (0.80 to 3.65)  p=0.16 | REF | 0.65  (0.44 to 0.95)  p=0.03 | 0.33  (0.19 to 0.56)  p<0.001 |
| **Overseas student** | 1.48  (0.59 to 3.74)  p=0.41 | 2.78  (1.15 to 6.74)  p=0.02 | REF | 1.37  (0.80 to 2.32)  p=0.25 | 1.82  (1.00 to 3.32)  p=0.05 |
| **Parents have higher education** | 1.19  (0.55 to 2.60)  p=0.66 | 1.01  (0.44 to 2.31)  p=0.98 | REF | 1.69  (1.10 to 2.58)  p=0.02 | 1.95  (1.13 to 3.32)  p=0.01 |
| **One or more disability** | 6.60  (2.23 to 19.6)  p=0.001 | 1.31  (0.59 to 2.87)  p=0.51 | REF | 0.36  (0.25 to 0.54)  p<0.001 | 0.22  (0.13 to 0.37)  p<0.001 |
| **BFI-2S: Agreeableness** | 0.88  (0.80 to 0.96)  p=0.003 | 1.06  (0.95 to 1.17)  p=0.29 | REF | 1.06  (1.01 to 1.11)  p=0.01 | 1.11  (1.05 to 1.18)  p=0.001 |
| **BFI-2S: Conscientiousness** | 0.99  (0.92 to 1.07)  p=0.75 | 1.09  (1.0o to 1.19)  p=0.06 | REF | 1.13  (1.09 to 1.18)  p<0.001 | 1.25  (1.19 to 1.33)  p<0.001 |
| **BFI-2S: Extraversion** | 0.99  (0.91 to 1.07)  p=0.77 | 1.02  (0.94 to 1.12)  p=0.60 | REF | 1.10  (1.05 to 1.14)  p<0.001 | 1.23  (1.17 to 1.29)  p<0.001 |
| **BFI-2S: Negative emotions** | 1.19  (1.06 to 1.34)  p=0.004 | 0.92  (0.84 to 1.02)  p=0.10 | REF | 0.81  (0.77 to 0.85)  p<0.001 | 0.70  (0.65 to 0.74)  p<0.001 |
| **BFI-2-S: Open mindedness** | 1.04  (0.95 to 1.13)  p=0.41 | 0.96  (0.87 to 1.05)  p=0.37 | REF | 1.01  (0.97 to 1.06)  p=0.63 | 1.09  (1.03 to 1.15)  p=0.002 |
| **Index of multiple deprivation** | 0.93  (0.81 to 1.07)  p=0.33 | 0.99  (0.84 to 1.18)  p=0.92 | REF | 1.09  (1.01 to 1.18)  p=0.03 | 1.04  (0.94 to 1.14)  p=0.47 |

**Table S4:** Results from univariable multinomial logistic regression models, with class 3 as the reference category. BFI-2-S: Big Five Inventory-2 short form. LGBT+: Lesbian, Gay, Bisexual, Transgender, other.

| **Variable** | **Class 1**  **(‘Strugglers’)** | **Class 2**  **(‘Improvers’)** | **Class 3**  **(‘Decliners’)** | **Class 4**  **(‘Constants’)** | **Class 5**  **(‘Thrivers’)** |
| --- | --- | --- | --- | --- | --- |
| **Male** | 1.10  (0.50 to 2.44)  p=0.81 | 0.33  (0.10 to 1.11)  p=0.07 | 1.11  (0.73 to 1.69)  p=0.62 | REF | 1.22  (0.79 to 1.89)  p=0.37 |
| **Non-white ethnicity** | 2.15  (0.88 to 5.25)  p=0.09 | 3.43  (1.43 to 8.28)  p=0.01 | 1.78  (1.01 to 3.11)  p=0.04 | REF | 1.65  (0.91 to 3.01)  p=0.10 |
| **LGBT+ sexuality** | 2.09  (1.08 to 4.05)  p=0.03 | 2.22  (1.08 to 4.57)  p=0.03 | 1.60  (1.10 to 2.34)  p=0.02 | REF | 0.57  (0.36 to 0.90)  p=0.02 |
| **Postgraduate student** | 1.42  (0.71 to 2.85)  p=0.33 | 1.33  (0.61 to 2.86)  p=0.47 | 0.78  (0.51 to 1.21)  p=0.27 | REF | 1.08  (0.70 to 1.68)  p=0.73 |
| **Previously sought help for a mental health condition** | 6.14  (2.76 to 13.6)  p<0.001 | 2.22  (1.07 to 4.61)  p=0.03 | 1.68  (1.17 to 2.42)  p=0.01 | REF | 0.69  (0.46 to 1.03)  p=0.07 |
| **Previously diagnosed with a mental health condition** | 7.19  (3.46 to 14.9)  p<0.001 | 2.64  (1.27 to 5.48)  p<0.001 | 1.54  (1.05 to 2.26)  p=0.03 | REF | 0.51  (0.31 to 0.83)  p=0.01 |
| **Overseas student** | 1.08  (0.46 to 2.56)  p=0.86 | 2.03  (0.90 to 4.61)  p=0.09 | 0.73  (0.43 to 1.24)  p=0.25 | REF | 1.33  (0.81 to 2.18)  p=0.25 |
| **Parents have higher education** | 0.71  (0.33 to 1.50)  p=0.37 | 0.60  (0.27 to 1.34)  p=0.21 | 0.59  (0.39 to 0.91)  p=0.02 | REF | 1.16  (0.71 to 1.89)  p=0.57 |
| **One or more disability** | 18.1  (6.22 to 52.6)  p<0.001 | 3.58  (1.67 to 7.68)  p=0.001 | 2.74  (1.84 to 4.07)  p<0.001 | REF | 0.60  (0.37 to 0.97)  p=0.04 |
| **BFI-2S: Agreeableness** | 0.83  (0.76 to 0.90)  p<0.001 | 0.99  (0.90 to 1.10)  p=0.91 | 0.94  (0.90 to 0.99)  p=0.01 | REF | 1.04  (0.99 to 1.10)  p=0.12 |
| **BFI-2S: Conscientiousness** | 0.87  (0.81 to 0.94)  p<0.001 | 0.96  (0.88 to 1.05)  p=0.35 | 0.88  (0.85 to 0.92)  p<0.001 | REF | 1.11  (1.06 to 1.16)  p<0.001 |
| **BFI-2S: Extraversion** | 0.91  (0.83 to 0.97)  p=0.01 | 0.94  (0.86 to 1.02)  p=0.11 | 0.92  (0.88 to 0.95)  p<0.001 | REF | 1.12  (1.07 to 1.17)  p<0.001 |
| **BFI-2S: Negative emotions** | 1.48  (1.31 to 1.66)  p<0.001 | 1.15  (1.05 to 1.25)  p=0.003 | 1.24  (1.18 to 1.30)  p<0.001 | REF | 0.86  (0.83 to 0.90)  p<0.001 |
| **BFI-2S: Open mindedness** | 1.03  (0.94 to 1.11)  p=0.55 | 0.95  (0.87 to 1.04)  p=0.25 | 0.99  (0.95 to 1.03)  p=0.63 | REF | 1.08  (1.03 to 1.13)  p=0.003 |
| **Index of multiple deprivation** | 0.85  (0.74 to 0.98)  p=0.03 | 0.91  (0.77 to 1.07)  p=0.26 | 0.92  (0.85 to 0.99)  p=0.03 | REF | 0.95  (0.87 to 1.04)  p=0.27 |

**Table S5:** Results from univariable multinomial logistic regression models, with class 4 as the reference category. BFI-2-S: Big Five Inventory-2 short form. LGBT+: Lesbian, Gay, Bisexual, Transgender, other.

| **Variable** | **Class 1**  **(‘Strugglers’)** | **Class 2**  **(‘Improvers’)** | **Class 3**  **(‘Decliners’)** | **Class 4**  **(‘Constants’)** | **Class 5**  **(‘Thrivers’)** |
| --- | --- | --- | --- | --- | --- |
| **Male** | 0.90  (0.39 to 2.08)  p=0.81 | 0.27  (0.08 to 0.94)  p=0.04 | 0.91  (0.56 to 1.49)  p=0.71 | 0.82  (0.53 to 1.27)  p=0.37 | REF |
| **Non-white ethnicity** | 1.30  (0.51 to 3.32)  p=0.58 | 2.08  (0.82 to 5.25)  p=0.12 | 1.08  (0.57 to 2.02)  p=0.82 | 0.61  (0.33 to 1.10)  p=0.10 | REF |
| **LGBT+ sexuality** | 3.68  (1.76 to 7.68)  p=0.001 | 3.91  (1.77 to 8.61)  p=0.001 | 2.81  (1.71 to 4.63)  p<0.001 | 1.76  (1.11 to 2.78)  p=0.02 | REF |
| **Postgraduate student** | 1.31  (0.62 to 2.77)  p=0.48 | 1.23  (0.54 to 2.77)  p=0.62 | 0.72  (0.43 to 1.21)  p=0.22 | 0.92  (0.60 to 1.43)  p=0.73 | REF |
| **Previously sought help for a mental health condition** | 8.94  (3.85 to 20.8)  p<0.001 | 3.24  (1.49 to 7.05)  p=0.003 | 2.45  (1.56 to 3.85)  p<0.001 | 1.46  (0.97 to 2.18)  p=0.07 | REF |
| **Previously diagnosed with a mental health condition** | 14.2  (6.27 to 32.2)  p<0.001 | 5.21  (2.30 to 11.8)  p<0.001 | 3.04  (1.79 to 5.18)  p<0.001 | 1.98  (1.20 to 3.25)  p=0.01 | REF |
| **Overseas student** | 0.81  (0.33 to 2.01)  p=0.65 | 1.53  (0.64 to 3.62)  p=0.34 | 0.55  (0.30 to 1.00)  p=0.05 | 0.75  (0.46 to 1.23)  p=0.25 | REF |
| **Parents have higher education** | 0.61  (0.27 to 1.39)  p=0.24 | 0.52  (0.22 to 1.23)  p=0.14 | 0.51  (0.30 to 0.87)  p=0.01 | 0.87  (0.53 to 1.42)  p=0.57 | REF |
| **One or more disability** | 30.3  (9.88 to 92.8)  p<0.001 | 5.99  (2.60 to 13.8)  p<0.001 | 4.59  (2.72 to 7.73)  p<0.001 | 1.67  (1.03 to 2.71)  p=0.04 | REF |
| **BFI-2S: Agreeableness** | 0.79  (0.72 to 0.87)  p<0.001 | 0.95  (0.86 to 1.06)  p=0.37 | 0.90  (0.85 to 0.96)  p=0.001 | 0.96  (0.91 to 1.01)  p=0.12 | REF |
| **BFI-2S: Conscientiousness** | 0.79  (0.72 to 0.86)  p<0.001 | 0.87  (0.79 to 0.95)  p=0.002 | 0.80  (0.75 to 0.84)  p<0.001 | 0.90  (0.86 to 0.95)  p<0.001 | REF |
| **BFI-2S: Extraversion** | 0.80  (0.74 to 0.87)  p<0.001 | 0.83  (0.76 to 0.91)  p<0.001 | 0.81  (0.77 to 0.86)  p<0.001 | 0.89  (0.85 to 0.93)  p<0.001 | REF |
| **BFI-2S: Negative emotions** | 1.71  (1.51 to 1.95)  p<0.001 | 1.33  (1.21 to 1.46)  p<0.001 | 1.44  (1.35 to 1.53)  p<0.001 | 1.16  (1.11 to 1.21)  p<0.001 | REF |
| **BFI-2S: Open mindedness** | 0.95  (0.87 to 1.04)  p=0.27 | 0.88  (0.80 to 0.97)  p=0.01 | 0.92  (0.87 to 0.97)  p=0.002 | 0.93  (0.88 to 0.97) | REF |
| **Index of multiple deprivation** | 0.90  (0.77 to 1.04)  p=0.16 | 0.96  (0.80 to 1.14)  p=0.62 | 0.96  (0.88 to 1.06)  0.47 | 1.05  (0.96 to 1.15)  p=0.27 | REF |

**Table S6** Results from univariable multinomial logistic regression models, with class 5 as the reference category. BFI-2-S: Big Five Inventory-2 short form. LGBT+: Lesbian, Gay, Bisexual, Transgender, other.

| **Classes** | **AIC** | **BIC** |
| --- | --- | --- |
| **1** | 20039.18 | 20085.58 |
| **2** | 19398.99 | 19473.23 |
| **3** | 19143.50 | 19245.58 |
| **4** | 19085.24 | 19215.16 |
| **5** | 19045.25 | 19203.00 |
| **6** | 19021.43 | 19207.02 |

**Table S7.** Information criteria for the latent profile models considered. AIC = Akaike information criterion, BIC = Bayesian information criterion.
